# Unexpected Extensive Hair Whitening Following Baricitinib Treatment for Alopecia Universalis: A Case Report and Mechanistic Insights

**DOI:** 10.7759/cureus.76287

**Published:** 2024-12-23

**Authors:** Abhesh Lawati Limbu, Jun Xie, Ji Q Song

**Affiliations:** 1 Department of Dermatology and Venereology, Zhongnan Hospital of Wuhan University, Wuhan, CHN

**Keywords:** alopecia universalis, baricitinib, hair whitening, jak inhibitor, melanogenesis, side effects

## Abstract

Alopecia universalis (AU) is a severe form of alopecia areata characterized by the complete loss of scalp and body hair. While Janus kinase (JAK) inhibitors like baricitinib have shown promise in promoting hair regrowth in severe cases of AU, unexpected side effects, such as hair depigmentation, have not been widely reported. We present the case of a young male with AU who experienced progressive and extensive whitening of his scalp and body hair following treatment with baricitinib. After one month of therapy, the patient observed both black and white hairs emerging, which gradually turned entirely white by the second month. Over the course of seven months, the whitening of the hair persisted, with no repigmentation observed. Laboratory tests and clinical evaluations indicated no significant adverse effects, suggesting that baricitinib was well tolerated. Dermoscopic examination revealed predominantly white terminal hairs. While the mechanisms underlying this phenomenon remain unclear, we discuss potential interactions between JAK inhibition and melanocyte function, suggesting that baricitinib’s modulation of the JAK-STAT pathway may impact melanogenesis and hair pigmentation. This case highlights the need for further investigation into the effects of JAK inhibitors on hair pigmentation and the potential for hair whitening as an uncommon side effect. Understanding these mechanisms is essential for improving treatment strategies for AU and addressing patient concerns regarding pigmentation changes during therapy.

## Introduction

Alopecia areata (AA) is a chronic, immune-mediated, nonscarring alopecia with a spectrum of severity, ranging from patchy or diffuse hair loss to more severe forms, such as alopecia totalis (AT), defined by complete scalp hair loss and alopecia universalis (AU), the most extreme form, involving complete loss of scalp and body hair [1]. In comparison to patchy AA, AT and AU are associated with greater inflammation, a more prolonged disease course, and increased treatment resistance, underscoring AU's severity as the most extensive and challenging manifestation of AA.

Alopecia areata affects individuals across all genders, ethnicities, and age groups, with a global prevalence of approximately 2% [2]. Among its more severe forms, AT has a prevalence of 0.08%, while AU is the rarest, affecting approximately 0.03% of the population [2]. AA is often associated with significant psychological impacts, including anxiety and depression, with suicides also reported, particularly among adolescents [1].

Research suggests that AA involves an autoimmune attack mediated by Th1 cells, CD8+ T cells expressing natural killer group 2D (CD8+NKG2D+ effector T cells), and inflammatory cytokines including interferon-γ (IFN-γ), interleukin-2 (IL-2), IL-7, and IL-15, along with type 2 cytokines like IL-4 and IL-13 [1,3]. Upregulation of IL-15 in hair follicles plays a key role in disrupting immune privilege by recruiting and activating CD8+NKG2D+ T cells, which promotes IFN-γ production and the upregulation of γ-chain cytokines. Furthermore, in AA, IFN-γ enhances IL-15 production in hair follicles via Janus kinase (JAK)1/2 signaling, creating a feedback loop that exacerbates CD8+NKG2D+ T cell infiltration and activation. Additionally, through JAK1/3 signaling, IL-15 further stimulates T-cell production of IFN-γ, amplifying the inflammatory response around hair follicles [1]. JAK inhibitors thus appear to be a viable therapeutic option for the treatment of AA.

Melanocytes, located in the hair bulb, play a critical role in the hair cycle by synthesizing melanin, the pigment responsible for hair color. During the anagen (growth) phase, melanocytes proliferate and migrate within the hair follicle, actively contributing to melanin production [4]. In contrast, during the catagen (regression) and telogen (resting) phases, melanocyte activity diminishes, leading to reduced melanin synthesis and potential depigmentation of the hair [4]. In AA, autoimmune targeting of melanocytes can disrupt this process, prematurely transitioning hair follicles from the anagen phase to catagen and telogen [4]. This disruption may contribute to hair whitening, a phenomenon occasionally observed during hair regrowth in AA patients.

Treatment options for AU remain limited. While corticosteroids (topical, intralesional, and systemic) and immunosuppressants (methotrexate, azathioprine, and cyclosporine) have been used off-label, their long-term efficacy is often unpredictable in severe AA cases, and these treatments are not approved by the Food and Drug Administration (FDA) [1]. JAK inhibitors, such as baricitinib, represent a promising targeted therapy. Baricitinib, a selective JAK1/JAK2 inhibitor and an FDA-approved oral medication for adults with severe AA, blocks the JAK-STAT pathway, thereby inhibiting downstream inflammatory signals such as IFN-γ and IL-15 [1]. This suppression reduces the activity and production of inflammatory T cells, helping to restore immune privilege at the hair follicle and facilitating the re-entry of hair follicles into the anagen phase [5]. Common side effects of baricitinib include upper respiratory infections, headache, nausea, and increased susceptibility to viral infections. Serious but less common risks involve elevated liver enzymes, lipid changes, and, rarely, thromboembolic events, as well as immunosuppression-related risks over prolonged treatment [1]. Notably, changes in hair color have not been reported as a direct side effect of baricitinib in the existing literature.

This case report highlights an unexpected and rare outcome of baricitinib treatment in a young male with AU, where significant hair regrowth was accompanied by gradual whitening of the scalp and body hair. To the best of our knowledge, no similar hair-whitening phenomenon has been directly associated with baricitinib in the existing literature. Understanding such atypical responses is crucial for improving treatment strategies in severe AA cases and highlights the need for further research into the complex interplay between JAK inhibitors and melanocyte function.

## Case presentation

**Figure 1:**
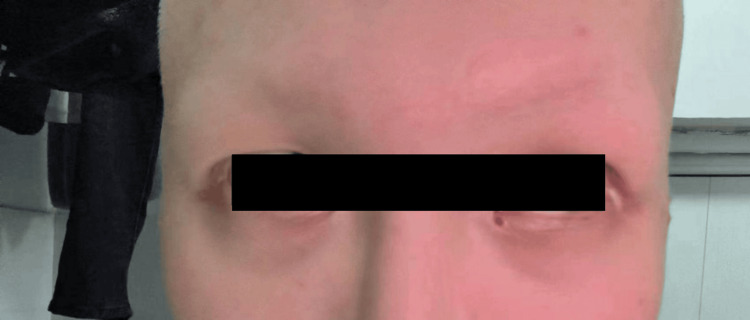
At admission, the patient presented with complete loss of scalp hair, eyebrows, and eyelashes.

A male in his early 20s presented with complete loss of scalp hair, eyebrows, and eyelashes over the past year (Figure 1). He reported no prior use of topical or systemic treatments for his condition. The patient was otherwise healthy and denied any personal or family history of AA or other autoimmune disorders such as thyroid disease or vitiligo. There was no history of stress, chemotherapy, or radiotherapy. The diagnosis of AU was confirmed based on a Severity of Alopecia Tool (SALT) score of 100 [6], reflecting complete scalp hair loss, along with clinical examination findings of total hair loss involving the scalp, eyebrows, eyelashes, axilla, and pubic regions.

Laboratory tests, including complete blood cell count, IgE levels, liver and kidney function tests, thyroid function tests, and screenings for hepatitis B, hepatitis C, syphilis, HIV, and tuberculosis (T-SPOT.TB), were conducted prior to initiating treatment with baricitinib (Table 1). The white blood cell (WBC) count was slightly elevated (10.76 × 10^9^/L; reference range: 4.1-11.0 × 10^9^/L), with an elevated neutrophil count (6.95 × 10^9^/L; reference range: 1.8-6.3 × 10^9^/L) and monocyte count (0.74 × 10⁹/L; reference range: 0.1-0.6 × 10⁹/L), suggesting a possible infection or inflammatory process. Direct bilirubin (DBIL) was elevated (8.3 μmol/L; reference range: 0-7 μmol/L), and cholinesterase (CHE) levels were also elevated (11,590 U/L; reference range: 3,000-10,500 U/L), potentially indicating subtle liver dysfunction, despite normal liver enzyme levels. Despite these mild deviations, no clinically significant laboratory abnormalities were identified that would contraindicate treatment. Baricitinib was initiated at a dose of 4 mg per day orally for the treatment of alopecia universalis.

**Table 1:** Lab results on admission and after two months of treatment.

SN	Test	Result (on admission)	Result (after 2 months)	Unit	Reference range
1	White blood cells (WBC)	10.76	7.70	10^9^/L	4.1–11.0
2	Red blood cells (RBC)	5.00	4.55	10^12^/L	4.3–5.8
3	Hemoglobin (HGB)	150.00	137.00	g/L	130–175
4	Platelet (PLT)	215.00	256.00	10^9^/L	125–350
5	Neutrophil percentage	64.60	67.70	%	37–77
6	Lymphocyte percentage	27.40	23.40	%	17–54
7	Monocyte percentage	6.90	5.80	%	2–11
8	Eosinophil percentage	1.00	2.60	%	0–9.0
9	Basophil percentage	0.10	0.50	%	0–1.0
10	Neutrophil count	6.95	5.21	10^9^/L	1.8–6.3
11	Lymphocte count	2.95	1.80	10^9^/L	1.1–3.2
12	Monocyte count	0.74	0.45	10^9^/L	0.1–0.6
13	Eosinophil count	0.11	0.20	10^9^/L	0.02–0.52
14	Basophil count	0.01	0.04	10^9^/L	0–0.06
15	Hematocrit (HCT)	44.90	40.60	%	39–51
16	Mean corpuscular volume (MCV)	89.80	89.20	fL	82–100
17	Mean corpuscular hemoglobin (MCH)	30.00	30.10	pg	25–34
18	Mean corpuscular hemoglobin concentration (MCHC)	334.00	337.00	g/L	316–354
19	Red cell distribution width (RDW)	12.20	12.30	%	10.1–16.0
21	Mean platelet volume (MPV)	10.00	10.40	fL	6–12
22	Complement C3	0.97	0.99	g/L	0.70–1.40
23	Complement C4	0.292	0.338	g/L	0.100–0.400
24	Immunoglobulin G (IgG)	10.30	10.70	g/L	8.60–17.40
25	Immunoglobulin A (IgA)	1.49	1.20	g/L	1.0–4.20
26	Immunoglobulin M (IgM)	1.60	1.25	g/L	0.3–2.20
27	Immunoglobulin E (IgE)	<18.4	<18.4	kU/L	0.0–100.0
28	Alanine transaminase test (ALT)	31	-	U/L	9–50
29	Aspartate transaminase test (AST)	12	-	U/L	15–40
30	AST/ALT ratio	0.39	-		0.2–2.0
31	Total billirubin (TBIL)	20.3	-	μmol/L	5–21
32	Direct billirubin (DBIL)	8.3	-	μmol/L	0–7
33	Indirect billirubin (IBIL)	12.0	-	μmol/L	1.5–18.0
34	Gamma glutamate transferase (GGT)	24	-	U/L	8–57
35	Alkaline phosphatase (ALP)	85	-	U/L	30–120
36	Total bile acid (TBA)	1.4	-	μmol/L	0–15
37	Cholinesterase (CHE)	11590	-	U/L	3000–10500
38	Blood urea nitrogen (BUN)	5.80	-	mmol/L	2.8–7.6
39	Creatinine	76.00	-	umol/L	64–104
40	Total protein (TP）	73.1	-	g/L	65–85
41	Albumin (ALB)	43.6	-	g/L	40–55
42	Globulin (GLB)	29.5	-	g/L	20–40
43	Albumin globulin ratio (A/G)	1.48	-		1.5–2.5
44	Uric acid (UA)	347.1	-	μmol/L	208–428
45	Carbon dioxide (CO_2_)	24.0	-	mmol/L	21–29
46	Cystatin C (CYSC)	0.96	-	mg/L	0.59–1.03
47	Potassium (K)	4.22	-	mmol/L	3.5–5.3
48	Sodium (Na)	135.00	-	mmol/L	137–147
49	Chlorine (Cl)	101.10	-	mmol/L	99–100
50	Calcium (Ca)	2.37	-	mmol/L	2.11–2.52
51	Phosphate (Phos)	1.04	-	mmol/L	0.85–1.51
52	Hepatitis B surface antigen (HBsAg)	0.00	-	IU/mL	0–0.05
53	Hepatitis C antibody (Anti-HCV)	0.06	-	s/co	0–1.00
54	HIV antigen/antibody (HIV-Ag/Ab)	0.10	-	s/co	0–1.00
55	Syphilis	0.05	-	s/co	0–1.00
56	Hepatitis C virus core antigen (HCV-cAg)	0.22	-	s/cAg	0–1.00
57	T-SPOT.TB test	Negative	-	-	Negative
58	Free triiodothyronine (FT3)	4.58	-	pmol/L	3.21–6.50
59	Free thyroxine (FT4)	18.40	-	pmol/L	10.20–21.88
60	Thyroid-stimulating hormone (TSH)	0.994	-	μIU/mL	0.3–4.6
61	Anti-thyroglobulin antibodies (Anti-Tg)	10	-	IU/mL	0–72
62	Anti-thyroid peroxidase antibodies (Anti-TPO)	<1.00	-	IU/mL	0–16
63	Vitamin D (VD)	52.326	-	nmol/L	25–200

**Figure 2:**
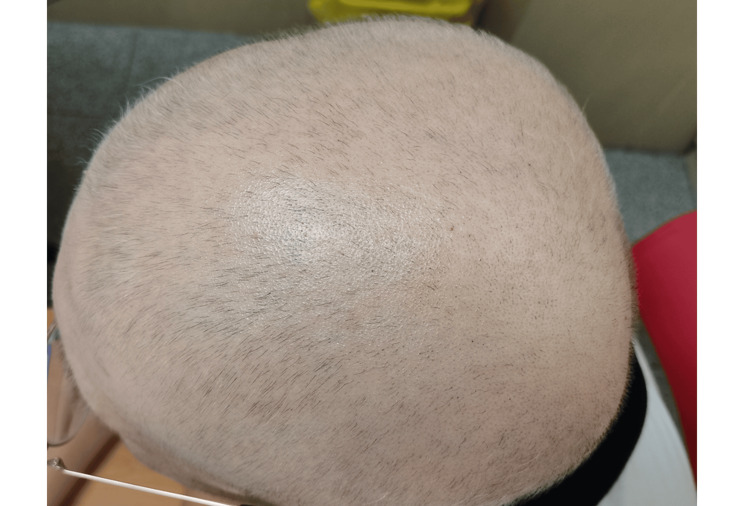
After one month of therapy, terminal black hairs with a few white hairs were observed in a diffuse pattern on the scalp.

Approximately two weeks after starting therapy, fine vellus hair began to grow on the scalp. At this stage, the patient opted to shave his scalp hair. After one month of treatment, terminal hair covered much of the scalp; however, the patient noticed a diffuse pattern of white hair emerging (Figure 2). This pattern suggested that some hairs were white from the onset of regrowth while others retained their pigmentation. Hair regrowth was also observed in the eyebrows, eyelashes, beard, and facial vellus hair.

**Figure 3:**
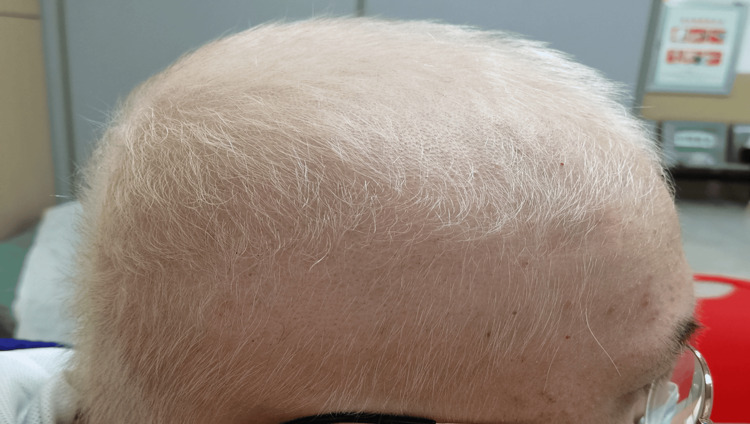
By second month of therapy, most of the patient's scalp hair had turned white and a few regrown eyebrows were also white.

By the second month of therapy, the majority of the patient's scalp hair had turned white, with only a few black hairs remaining. Some regrown eyebrows had also turned white (Figure 3). Laboratory tests conducted after two months of treatment were used to monitor for potential side effects of baricitinib (Table 1).

The WBC count, which was 10.76 × 10⁹/L on admission, decreased to 7.70 × 10⁹/L, remaining within the normal reference range (4.1-11.0 × 10⁹/L). Similarly, the neutrophil count decreased from 6.95 × 10⁹/L (previously elevated) to 5.21 × 10⁹/L, now within the normal range (1.8-6.3 × 10⁹/L). The monocyte count also decreased from 0.74 × 10⁹/L (elevated) to 0.45 × 10⁹/L, falling within the normal range (0.1-0.6 × 10⁹/L). These trends indicate no significant changes and suggest the absence of systemic inflammation or infection. Other hematologic parameters, including hemoglobin, red blood cell (RBC) count, and platelet levels, remained within normal limits, indicating the absence of adverse hematologic effects.

IgG levels showed a slight increase (from 10.30 g/L to 10.70 g/L) within the normal range (8.60-17.40 g/L), indicating preserved humoral immunity. In contrast, IgA and IgM levels exhibited mild reductions (from 1.49 g/L to 1.20 g/L for IgA and from 1.60 g/L to 1.25 g/L for IgM). While these changes remained within the reference ranges, they may reflect minor variations potentially associated with baricitinib's modulation of cytokines and its impact on immune cell dynamics. IgE levels remained unchanged, indicating no evidence of hypersensitivity or allergic responses.

**Figure 4:**
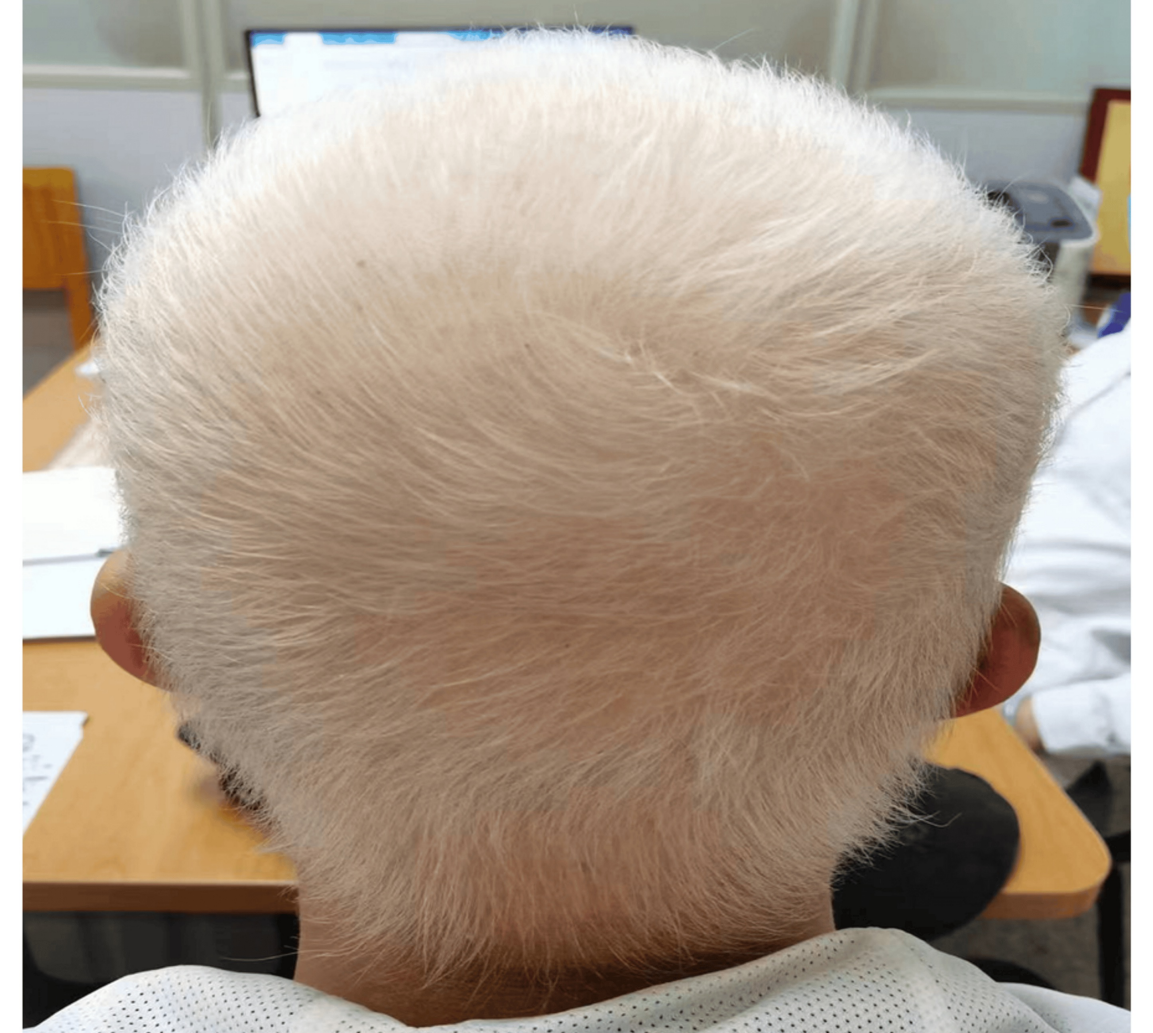
After seven months, all scalp hair turned white.

After approximately seven months of therapy, all regrown scalp hair (Figure 4), as well as the eyebrows, eyelashes, beard, armpit hair, pubic hair, and vellus hair, gradually turned white. This hair whitening appears to be a progressive and likely permanent phenomenon rather than temporary hypopigmentation, as no repigmentation was observed throughout the treatment period. While the treatment was well-tolerated with no adverse effects reported, the progressive whitening of the hair may represent an unexpected side effect of baricitinib.

**Figure 5:**
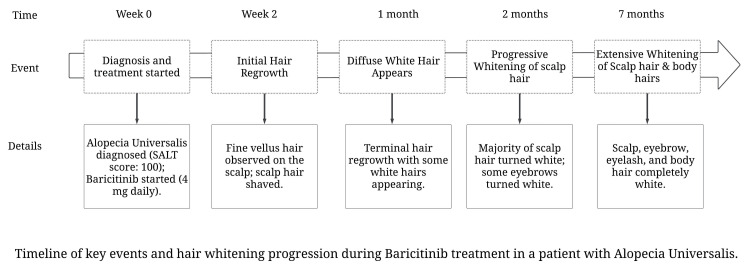
Timeline of key events and hair whitening progression during baricitinib treatment in a patient with alopecia universalis.

Figure 5 illustrates the progression of key events and hair whitening during baricitinib treatment in a patient suffering from alopecia universalis.

**Figure 6:**
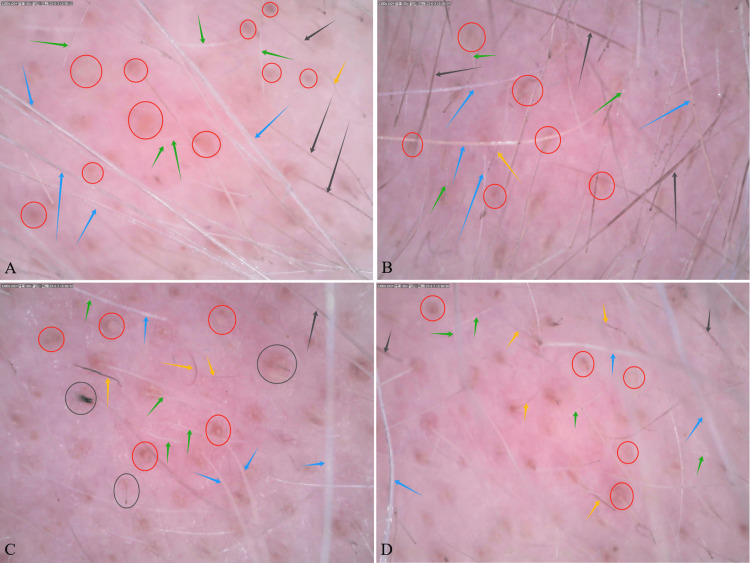
Hair dermoscopy at ten months revealed predominantly white terminal hairs (blue arrow), a few depigmented black terminal hairs (black arrow), vellus white hairs (green arrow), and vellus depigmented black hairs (yellow arrow). The scalp appeared pinkish-white with multiple yellow dots (red circle) and broken black hairs in the right frontotemporal region (black circle). Images show (A) vertex, (B) anterior hairline, (C) right frontotemporal, and (D) left frontotemporal views. Dermoscopy was performed with a Dino-Lite digital microscope at 50× magnification.

Hair dermoscopy at ten months revealed predominantly white terminal hairs (blue arrows), along with a few depigmented black terminal hairs (black arrows), vellus white hairs (green arrows), and vellus depigmented black hairs (yellow arrows) in the vertex (Figure 6A), right frontotemporal (Figure 6C), and left frontotemporal (Figure 6D) regions of the scalp. In contrast, the anterior hairline (Figure 6B) showed a mixed pattern, with an approximately equal amount of white terminal hairs (blue arrows) and depigmented black terminal hairs (black arrows), along with a few vellus white hairs (green arrows) and vellus depigmented black hairs (yellow arrows). The scalp appeared pinkish-white across all regions, with multiple yellow dots (red circles) noted in each view. Notably, broken black hairs (black circles) were observed only in the right frontotemporal region (Figure 6C), while exclamation mark hairs were absent across all regions (Figure 6A-6D).

## Discussion

This case report documents a young male with AU who initially experienced hair regrowth following treatment with baricitinib, followed by gradual and widespread whitening of hair. To the best of our knowledge, such widespread hair whitening of scalp hair, eyebrows, eyelashes, armpit hair, pubic hair, and vellus hair following baricitinib treatment for AU remains previously undocumented.

It is known that the regrowth of hair within a patch of alopecia areata is often depigmented, which can be temporary or persistent [7]. Most cases typically involve temporary hypopigmentation that lasts no longer than the duration of the first regrowth cycle before normal pigmentation resumes [8-10]. In our case, approximately one month after starting baricitinib, we observed a combination of predominantly black hairs with a few white hairs (as shown in Figure 2). The patient then noticed that the majority of hairs turned white within two months of baricitinib treatment (as shown in Figure 3). After approximately seven months of therapy, all regrown scalp hair (as shown in Figure 4), as well as the eyebrows, eyelashes, beard, armpit hair, pubic hair, and vellus hair, gradually turned white. This aligns with the observation that AA preferentially targets pigmented hair follicles while sparing white hairs [7,8]. Jia et al. reported a case series in which only pigmented hairs were affected, while gray hairs were spared in the alopecia areata patches [7]. Similarly, Tan et al. reported a 47-year-old male with AA who lost only black hair in the affected patches without involvement of regrowing white hair [8].

Persistent hypopigmented hair regrowth after AA treatment has been reported by Wade and Sinclair in a 17-year-old girl with a persistent patch of white hair lasting seven years [9]. Dinh and Chong also reported a similar case in a 62-year-old woman whose widespread non-pigmented hair regrowth persisted for six months after prednisolone treatment for diffuse AA [10]. Additionally, Tian et al. reported two cases of persistent white hair regrowth after AA, which eventually regained pigmentation following prolonged treatment (57 and 39 months) with glycyrrhizin and methylprednisolone [11]. In our case, the whitening persisted throughout the 10-month treatment period. We propose that the observed white hairs were not only spared but also actively regenerated on the scalp, as reported by Tian et al. [11]. However, in our case, there was evidence of gradual whitening not only of the scalp hair but also of the eyebrows, eyelashes, and body hair (Table 2).

**Table 2:** Summary of reported cases of persistent white hair following treatments for alopecia areata.

Study	Patient Characteristics	Treatment Used	Duration of Depigmentation	Reversibility
Wade and Sinclair [9]	17-year-old female initial occipital hair loss regrew as white hair; temporal AA patches with exclamation mark hairs regrew pigmented hair following treatment.	Diphenylcyclopropenone (DCP) sensitized with 2% solution, treated with 0.005–0.01% weekly	Persistent, 7 years, occipital area	Not reported
Dinh and Chong [10]	62-year-old female, diffuse AA with >50% scalp hair loss sparing non-pigmented hairs, active over 4 months, regrew as non-pigmented hair after treatment. Eyebrows did not regrow.	Oral prednisolone (37.5 mg/day, tapered over 6 weeks)	Sustained non-pigmented regrowth at 6 months	Not reported
Tian et al., case 1 [11]	47-year-old female, diffuse AA, initial regrowth of white hair across the scalp after shedding, later progressing to black hair upon treatment.	Initial: trenbolone (3 months, stopped). Later: methylprednisolone (8 mg/day) and compound glycyrrhizin tablets (50 mg/day).	The majority of white hair transitioned to black after 18 months, with only a small area remaining white (3 cm × 3 cm), complete repigmentation by 57 months	Fully reversible, with complete black regrowth confirmed via telephone
Tian et al., case 2 [12]	7-year-old male, diffuse AA with initial sparse white hair throughout the scalp.	Methylprednisolone (4 mg/day) and compound glycyrrhizin tablets (50 mg/day)	Transitioned to black hair after 12 months; complete black regrowth by 39 months	Fully reversible, with complete black regrowth

The selective retention of white hair in AA can also be observed in canities subita, commonly known as Marie Antoinette syndrome or the "overnight graying" phenomenon. This condition involves the sudden whitening of hair due to an acute onset of diffuse AA, often triggered by extreme stress or trauma. It typically presents as a "salt-and-pepper" pattern of hair pigmentation [12]. The historical case of French queen Marie Antoinette, whose hair reportedly turned gray overnight at age 38, just before facing the guillotine, is widely known [12]. Tan et al. reported a more clinically relevant example of this phenomenon in modern times, describing an 82-year-old woman who experienced sudden hair whitening within days and later received a diagnosis of diffuse AA [13]. However, a recent study on mice revealed that acute stress activates the sympathetic nervous system, leading to the depletion of melanocyte stem cells. This process is independent of immune attacks or adrenal stress hormones and is instead driven by norepinephrine released from sympathetic nerves [14]. In our case, with ongoing treatment over approximately 10 months as the primary intervention and no evidence of extreme stress or trauma, canities subita seems unlikely. Furthermore, the distinct selective loss of pigmented hair, combined with the absence of skin hypopigmentation, rules out vitiligo. Negative syphilis and HIV test results exclude the possibility of diffuse syphilitic alopecia and HIV-induced diffuse alopecia. Finally, the lack of any triggering illness or stress, along with the selective loss of pigmented hairs, reduces the likelihood of telogen effluvium.

Histopathological findings from previous cases of white hair regrowth in AA suggest that this phenomenon results from defective melanocyte activity rather than melanocyte depletion [15]. Hair follicle melanocytes, although present, display reduced numbers, lower melanin content, and impaired pigment transfer to cortical keratinocytes during the anagen phase [15]. Studies by Tobin et al. provided the first evidence that hair follicle melanocytes are specifically targeted in AA. Their findings showed that antibodies present in AA serum preferentially target antigens expressed by hair follicle melanocytes [16]. Gilhar et al. demonstrated that melanocyte-associated peptides can activate lesional T cells, inducing AA-like lesions [17]. In a similar study, Nagai et al. induced AA-like lesions in mice by inducing CD8+ T-cell-mediated immunity, specifically targeting hair follicle melanocytes [18]. Bertolini et al. provided visual evidence of T cells recognizing autoantigens presented by melanocytes and attacking them within hair follicles [19]. Although the specific autoantigen responsible for AA has not been identified, several studies suggest that peptides like MAGE-A3, Melan-A/Mart-1, gp100, gp100-derived peptides (G9-209, G9-280), PMEL17, and MC1R may play a role in triggering autoimmune attacks that lead to AA in mice [17,20].

Research has suggested a possible pathogenic role of the Th2 axis in AA, with elevated levels of Th2-related cytokines observed in AA patients [21]. Studies indicate that IL-4, a Th2 cytokine, can inhibit melanogenesis by downregulating the transcription and translation of MITF and dopachrome tautomerase via the JAK2-STAT6 signaling pathway [22]. While IL-4 does not directly suppress melanin synthesis, it may interact with other cytokines to modulate melanocyte activity. This suggests that the complex cytokine milieu in AA could influence both melanocyte function and hair pigmentation. In contrast, IL-10 does not significantly impact melanogenesis but may play a protective role for melanocytes [23]. On the other hand, IL-13 directly suppresses melanogenesis by reducing tyrosinase and dopachrome tautomerase levels through the JAK2-STAT6 pathway [24]. We did not perform cytokine testing in our case. However, IgE, IgA, IgM, and IgG levels were within normal ranges in our patient, potentially indicating the absence of significant systemic inflammation. Given baricitinib’s role as a JAK1/2 inhibitor and its potential to modulate Th2-related cytokine signaling, it is plausible that its effects on the IL-4 and IL-13 pathways may have influenced melanogenesis, contributing to hair depigmentation. However, this hypothesis remains speculative, and further research is needed to elucidate the specific mechanisms by which JAK inhibitors impact hair pigmentation.

Baricitinib's direct role in causing hair whitening remains unclear. JAK inhibitors primarily target immune responses and are not known to directly affect melanin production. However, the JAK-STAT signaling pathways are complex and exhibit crosstalk among different JAKs [25]. Inhibiting JAK1/2 with baricitinib could potentially have unintended consequences on other JAK pathways due to this crosstalk. Interestingly, a single case report documented the development of vitiligo - a depigmenting condition - in a rheumatoid arthritis patient treated with Tofacitinib, a JAK1/3 inhibitor [26]. Another case report described a paradoxical increase in cytokines, including IL-4, IL-6, IL-10, IFN-γ, and IL-17, after six months of Tofacitinib treatment in a patient with alopecia universalis [27]. Conversely, baricitinib has demonstrated repigmenting effects in patients with vitiligo, highlighting its potential role in melanogenesis - the biological process responsible for pigment production in skin and hair [28]. This duality-repigmentation in some cases and potential depigmentation in others - raises intriguing questions about its precise effects on melanocyte function and pigmentation pathways, underscoring the need for further investigation. Further research is specifically needed to understand how baricitinib affects the IL-4 and IL-13 signaling pathways in the context of melanogenesis and whether it influences hair pigmentation in cases like this one. While treatments like JAK inhibitors promote hair growth, they may not fully restore melanocyte function, potentially leading to depigmented regrowth. Further research is needed to understand these processes better and develop therapies addressing both hair regrowth and pigmentation in AA.

## Conclusions

This case report highlights a rare and unexpected outcome of baricitinib treatment in a young male with alopecia universalis, in which progressive and extensive whitening of scalp and body hair occurred. While baricitinib demonstrated its therapeutic potential by promoting significant hair regrowth, the whitening of newly grown hair raises important questions about the interplay between JAK-STAT pathway modulation and melanocyte function. This potential side effect may be distressing for some patients, emphasizing the need for clinicians to provide thorough counseling regarding its implications and to monitor pigmentation changes throughout treatment. The absence of prior reports on this phenomenon suggests that it may be rare and underexplored, warranting further investigation into baricitinib’s impact on melanogenesis. Future research should focus on understanding cytokine profiles, melanocyte activity, and molecular pathways involved to determine whether this phenomenon is transient or persistent and to refine therapeutic strategies for managing alopecia universalis.
